# Bifocal femoral lengthening assisted by preoperative 3-dimensional design in the restoration of posttraumatic limb length discrepancy

**DOI:** 10.1186/s12893-022-01697-7

**Published:** 2022-06-27

**Authors:** Maimaiaili Yushan, Yimurang Hamiti, Ainizier Yalikun, Cheng Lu, Aihemaitijiang Yusufu

**Affiliations:** Department of Microrepair and Reconstructive Surgery, The First Affiliated Hospital of Xinjiang Medical University, Ürümqi, Xinjiang People’s Republic of China

**Keywords:** Femur, Ilizarov technique, Limb lengthening, Limb length discrepancy, Trauma

## Abstract

**Background:**

To assess the clinical outcomes of preoperative three-dimensional planning followed by bifocal femoral lengthening in the treatment of posttraumatic limb length discrepancy (LLD).

**Methods:**

A total of 8 eligible patients with posttraumatic femoral LLD > 6 cm were admitted to our institution from January 2015 to January 2018 and treated by bifocal femoral lengthening with the assistance of 3-dimensional imaging technology. The following data were collected: detailed demographic information, the amount of lengthening, external fixation time (EFT), external fixation index (EFI), postoperative bone and functional outcomes, and complications in the follow-up period.

**Results:**

All included patients were successfully followed up for in an average of 55.4 ± 6.7 months after removal of the external fixator. There were six males and two females with an average age of 38.4 ± 12.2 years. The mean preoperative LLD was 69.2 ± 6.2 mm. The mean lengthening amount was 67.5 ± 6.9 mm. The mean EFT was 180.1 ± 20.2 days. The EFI was 26.73 ± 1.36 days/cm on average. All patients achieved satisfactory postoperative bone and functional outcomes. No major complications such as nerve or vascular injury were observed.

**Conclusions:**

Bifocal femoral lengthening with preoperative three-dimensional design provided precise surgical guidance and resulted in satisfactory postoperative outcomes, demonstrating that it is an effective treatment for posttraumatic femoral LLD.

## Introduction

The management of limb length discrepancy (LLD) continues to be a challenging subject in orthopedics, especially when LLD is secondary to trauma, owing to its long course and unpredictable clinical outcome [1–4]. Limb lengthening was an essential treatment for the conditions mentioned above, which was first described by Alessandro Codivilla and further developed in the twentieth century [4, 5].

Currently, the Ilizarov technique based on distraction osteogenesis is the cornerstone of all bone lengthening procedures [5–7]. Ilizarov’s principle was applied to treat a wide variety of conditions such as bone shortening, bone defects, nonunion, fracture, and deformities [8]. Despite its unique ability to produce osteogenesis and regeneration, this prolonged procedure is restricted by the undesirable long duration of treatment and associated complications. To overcome the inevitable long period required for external fixation with monofocal limb lengthening, studies using bifocal limb lengthening (to shorten the total treatment time by distracting two osteotomy sites and increasing the daily lengthening amount), lengthening over a nail, lengthening followed by nailing or plating, intramedullary nails without the need for external fixation, and recently, motorized internal lengthening nails PRECISE and STRYDE have been reported with successful outcomes [9–13].

In this study, based on a summary of the previous method and our experience, we adopted a the three-dimensional design for precise preoperative planning and combined it with bifocal femoral lengthening, treating eight patients with posttraumatic limb length discrepancy. The clinical outcomes of all included patients were presented to provide the efficacy of this combined technique in the restoration of posttraumatic LLD.

## Patient and method

This was a retrospective, single-center, therapeutic study that included patients with posttraumatic femoral LLD > 6 cm who were treated by bifocal femoral lengthening with the assistance of preoperative 3-dimensional imaging technology design between January 2015 and January 2018. The Institutional Ethics Committee of our institution approved the study protocol, and the informed consent was obtained from all participants. Patients who were under the age of 18 or had inadequate follow-up data were excluded from the study. Overall, eight patients were included in the present study. The following detailed data were obtained and recorded from medical files: age, gender, injury mechanism, preoperative LLD, amount of lengthening, external fixation time (EFT), external fixation index (EFI), postoperative bone and functional outcomes, and complications in the follow-up period. A minimum follow-up of 2 years was achieved for all patients after removal of the external fixator.

### Preoperative preparation

**Fig. 1:**
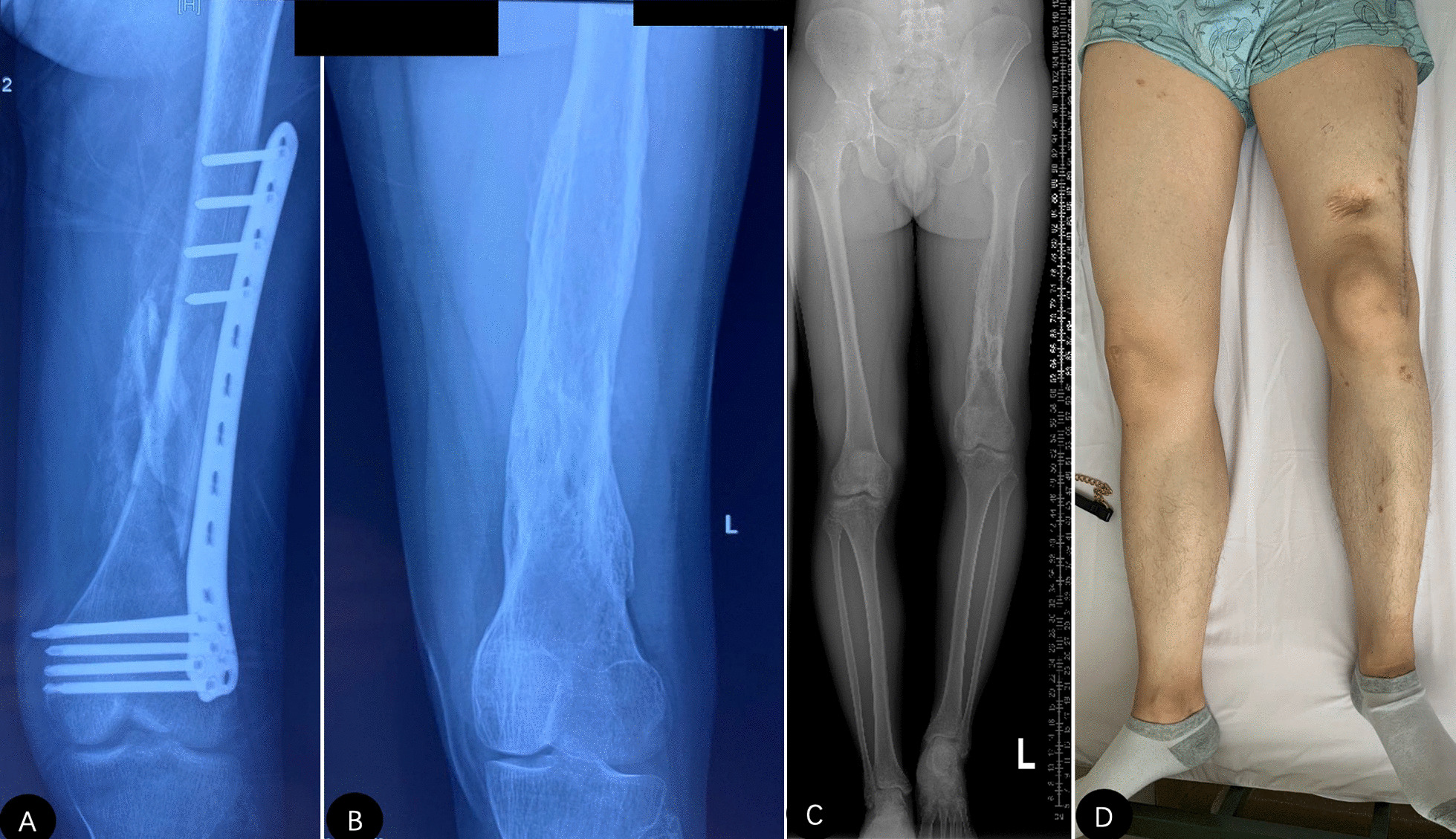
** A** A 22-year-old male patient was initially treated with limb shortening and plate fixation for the left femoral fracture (GA-type 3 C) caused by a traffic accident at local hospital. **B–D** Preoperative radiographs and general appearance showed limb length discrepancy and massive heterotopic ossification around distal femur

**Fig. 2:**
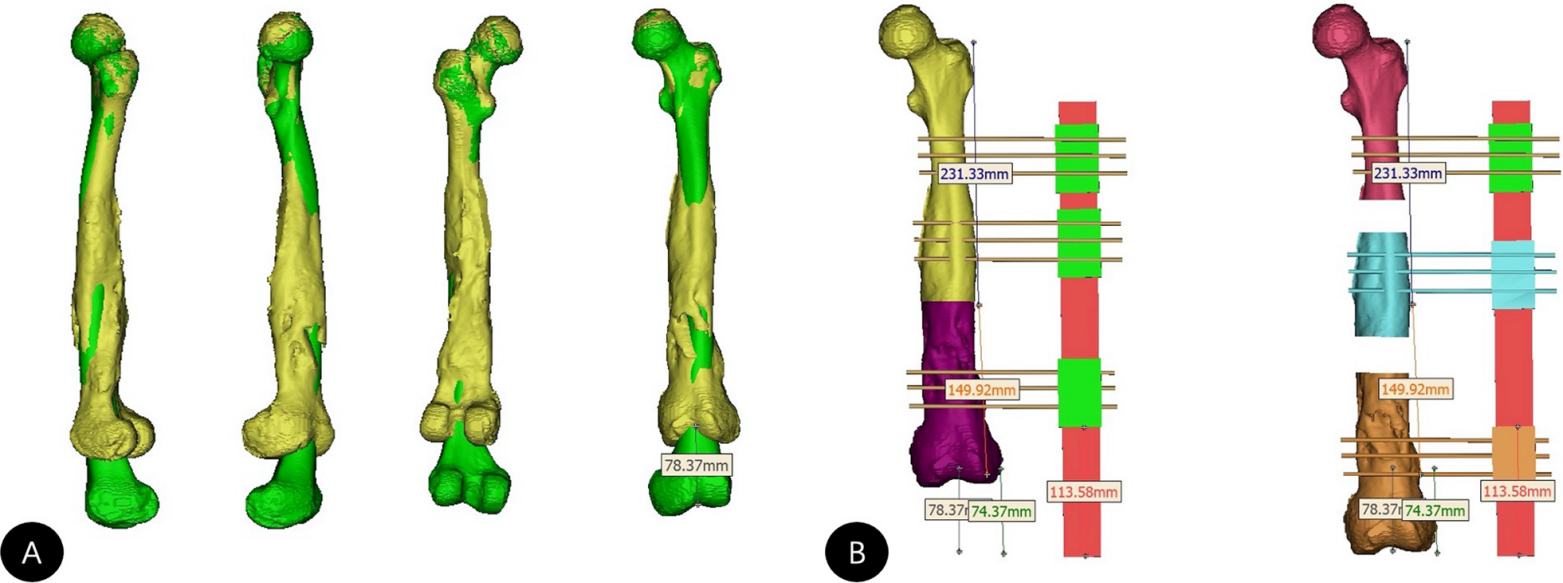
** A** Comparison and overlap of left and right femurs using 3-dimensional reconstruction images in different colors to calculate the exact LLD. **B** Simulation of the installation of external fixation, the osteotomy sites and limb lengthening process

It was necessary to undertake a preoperative physical examination, clinical laboratory test, and DSA (digital subtraction angiography) to determine whether the patient had any residual bone and soft tissue infections and vessel injury from previous trauma to determine whether the patient was suitable for limb lengthening. Precise preoperative planning was performed with the computerized tomography scan for the following objectives (Figs. 1 and 2): to obtain a three-dimensional reconstruction model from both the patient’s right and left lower extremities by transmitting the scanned image data to the workstation, then analyzing the data by the image processing software, and reorganizing the multiplane. The data were then imported into the modeling software (Materalise, version 17.0.0.435, Leuven, Belgium). An image of the patient’s healthy lower extremity was used to produce a digital model for the reference femur. The image was altered using the “mirror” tool in the software to create a model and performed the best fitting of the femur to guide the restoration of the patient’s short lower extremity; to identify the extent of the abnormal bone marrow cavity to calculate the distance of the clamps, screws and osteotomy sites (avoid potential injury to the femoral nutritive artery) and ultimately to obtain the optimal position of the external fixator; and to calculate the exact amount of LLD and assess the deviation of the mechanical axis to determine whether limb lengthening is applicable.

### Surgical technique

**Fig. 3:**
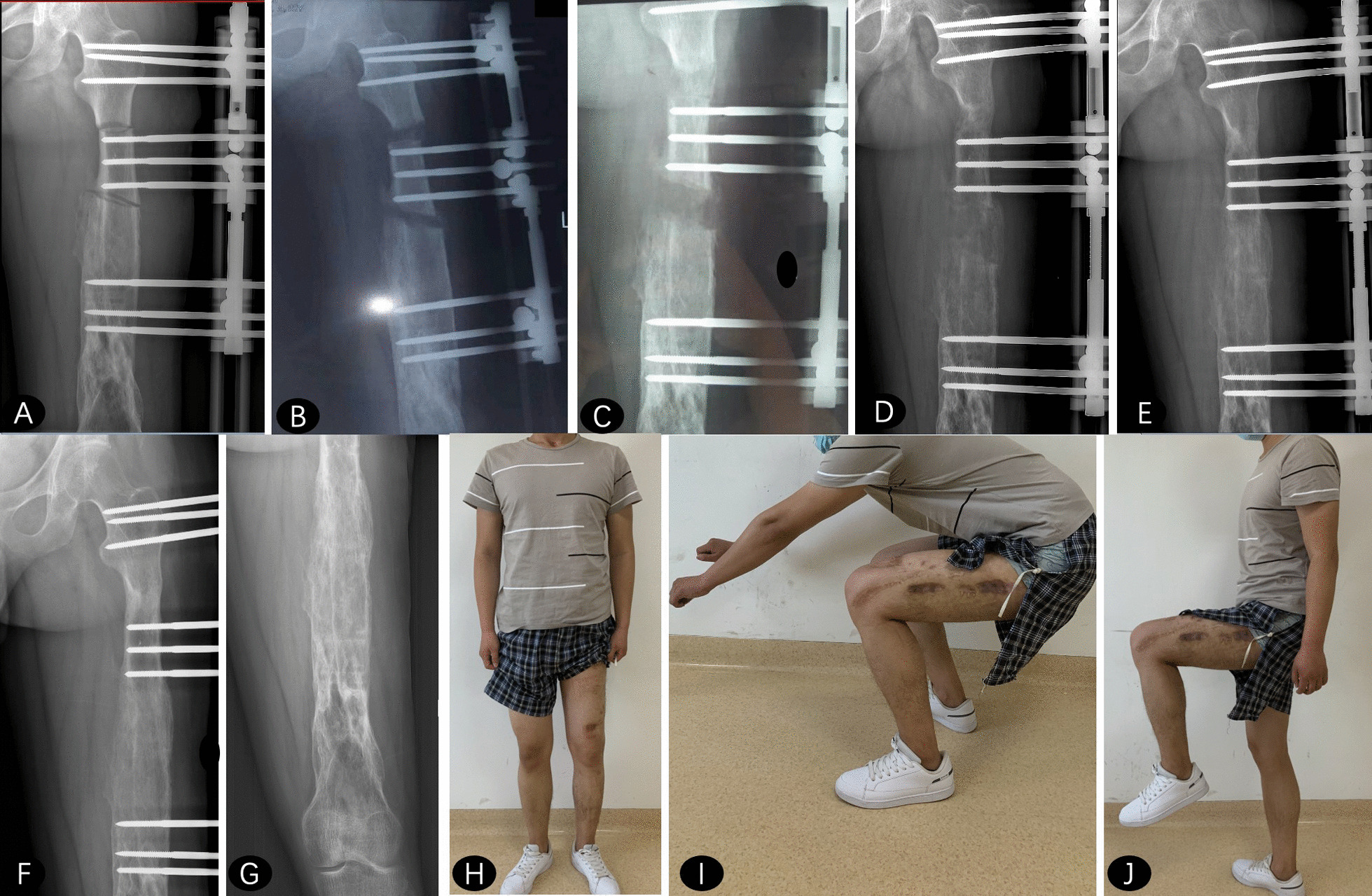
** A–E** Distraction at both osteotomy sites was started and evaluated by standard X ray at different time point. **F** Bifocal femoral lengthening was completed with good regenerate consolidation at 6 months after surgery. **G** External fixator was removed with excellent bone result assessed by ASAMI system. **H–J** General appearance at last visit with the excellent functional outcome

Under general or spinal anesthesia, patients were placed in the supine or lateral position and underwent bifocal limb lengthening using monolateral external fixation (Orthofix LRS, Shanghai CIIC Medical Instrument Co., Ltd). The external fixator was place on the anterolateral side of the thigh, which should be parallel to the mechanical axis of the femur. Schanz screws were inserted at the appropriate level in accordance with the preoperative 3-dimensional design. There were at least three Schanz screws located in each clamp with the necessary distance to stabilize the bone segment adequately. To achieve optimal treatment outcomes, all Schanz screws were inserted perpendicular to the femoral mechanical axis and across the diameter of the bone. Thermal necrosis was avoided by a slower drilling speed and constant saline irrigation. According to the results of the preoperative CT scan, the aberrant bone marrow cavity was avoided, and a minimally invasive percutaneous osteotomy was performed on the femur with the Gigli saw at preselected osteotomy sites (bifocal). It is crucial to prevent damage to the femoral nutritive artery during osteotomy (Fig. 3).

### Postoperative management

Lengthening started at both osteotomy sites after the latency period of 7 days with a rate of 1.0 mm/day at the metaphyseal site and 0.75 mm/day at the diaphyseal site due to the slower regenerated formation of the diaphyseal site and the soft tissue response. Immediate partial weight-bearing and passive knee exercises were commenced following surgery. Regular pin site care was performed with the appropriate care protocol. It was essential to execute physiotherapy regimens to control the range of motion of the joints and progressively restore the weight-bearing capacity. If a substantial loss of range of motion was observed during the lengthening phase, the lengthening rate was reduced until joint mobility was restored. All of the patients who took part in the study were followed up on a regular basis. The clinical and radiological evaluations of all patients were performed every 2 weeks to assess the lengthening process and bone regeneration, and any necessary measures were taken to prevent problems. Gradually, weight-bearing exercises were resumed during the consolidation phase of the procedure. The external fixator was removed after bone union was achieved, which was defined as at least three bridging callus appearing on anteroposterior and lateral radiographs [14]. Additionally, quantitative computerized tomography (QCT) was performed to measure regenerated consolidation in order to compensate for the variability in radiographic findings.

### Outcome evaluation

The following outcome measurements were evaluated through electronic medical records, while imaging was obtained from the hospital picture archiving and communication system: preoperative LLD, the amount of lengthening, EFT, EFI, postoperative bone and functional outcomes, and complications in the follow-up period. The amount of time (days) spent in the external fixator was known as EFT. The EFI was calculated by dividing the time (days) in the EFT by the lengthening achieved (cm). The Association for the Study and Application of the Method of Ilizarov (ASAMI) grading system was used to evaluate the bone and functional outcomes of the participants [8]. Complications that occurred throughout the therapy period were documented and classified as problems, obstacles, or true complications using Paley’s classification, which were resolved by nonsurgical and surgical intervention before the end of treatment or remained unresolved at the end of therapy [15].

## Results

There were 8 patients including 6 males and 2 females with posttraumatic LLD, and the mean age was 38.4 ± 12.2 years (range, 22–57 years). After the external fixator was removed, a minimum of 2 years of follow-up was achieved for all patients, and the mean follow-up was 55.4 ± 6.7 months (range, 48–67 months). There were 3 cases of falls and 5 cases of traffic accidents among the injury mechanisms. The mean preoperative LLD was 69.2 ± 6.2 mm (range, 61.8–78.4 mm), with all of the lesions located in the distal femur. The mean amount of lengthening achieved was 67.5 ± 6.9 mm (range, 58.5–78.4 mm). The average duration of external fixation was 180.1 ± 20.2 days (range, 148–205 days), while the average EFI was 26.73 ± 1.36 days/cm (range, 25–28.5 days/cm). The above results are summarized in Table 1. Bone and functional outcomes were assessed using the ASAMI criteria, and the results are given in Table 2. The bone outcomes were excellent in 5, good in 2, and fair in 1 case, and functional outcomes were excellent in 4, good in 3, and fair in 1 case. Complications throughout the therapy period were documented and classified using Paley’s classification and are shown in Table 3. Overall, there were 27 complications, which included 13 problems, 8 obstacles, and 6 true complications. Pin site infection occurred in 7 patients, which also indicates that it is the most common complication. Complications including nerve or vascular injury were not observed in this research.

**Table 1:** Demographic data of 8 patients

Case	Age/Gender	Injury mechanism	Preoperative LLD (mm)	Lengthening achieved (mm)	TIDL (days)	EFT (days)	EFI (days/cm)	Follow-up period (months)
1	22/M	Traffic accident	78.4	78.4	39.2	205	26.1	51
2	51/F	Fall	62.5	59.3	31.3	148	25.0	62
3	31/M	Traffic accident	67.1	67.1	33.6	179	26.7	48
4	45/M	Traffic accident	73.5	73.5	36.8	193	26.3	53
5	57/M	Fall	64.2	64.2	32.1	161	25.1	67
6	41/M	Traffic accident	74.7	71.3	37.4	201	28.2	59
7	33/M	Traffic accident	71.3	67.6	35.7	188	27.9	49
8	27/F	Fall	61.8	58.5	30.9	167	28.5	54

*LLD* limb length discrepancy, *EFT* external fixation time, *EFI* external fixation index, *M* male, *F* female, *TIDL* time interval of designed lengthening

**Table 2:** Evaluation of the bone and functional results according to ASAMI classification

Outcomes	Numbers/Percentage		
	Excellent	Good	Fair
Bone results	5 (62.5%)	2 (25%)	1 (12.5%)
Functional results	4 (50%)	3 (37.5%)	1 (12.5%)

**Table 3:** Complications according Paley criteria

Parameter	Problems	Obstacles	Complications	Total
Muscle contraction	3	1	0	4
Axial deviation	0	1	2	3
Delayed consolidation	1	3	0	4
Pin problems	7	0	0	7
Repeat fracture	0	0	0	0
Joint stiffness	1	1	4	6
Other	1	2	0	3
Total	13	8	6	

## Discussion

As mentioned in the literature, posttraumatic LLD is a complex deformity that significantly impairs patients’ quality of life, and there is no standard method of restoration treatment, necessitating the orthopedic surgeon to choose a treatment approach based on their experience and the patient’s objective condition, which presents a number of challenges that adversely affect clinical outcomes [1–7]. To treat the aforementioned conditions, limb lengthening with a ring or monolateral external fixator is a commonly used surgical technique with good therapeutic results. However, many studies have concluded that external fixators are undesirable due to their lengthy application time, which can effortlessly lead to complications such as pin site infection and knee stiffness. Other procedures and equipment, such as limb lengthening with high distraction rates, bifocal limb lengthening, intramedullary nails without the need for external fixation, and motorized internal lengthening nails, have been developed to decrease the external fixation duration [1, 3–5, 9, 16]. However, restoration of posttraumatic limb length discrepancy is not only conducted in major hospitals, and many primary hospitals do not adopt the abovementioned new equipment due to the high costs. Thus, there is an urgent need to evaluate a novel surgical procedure to achieve satisfactory clinical outcomes with traditional instruments that are less expensive than newer devices. Bifocal femoral lengthening assisted by preoperative 3-dimensional design provides one possibility.

Enhancing distraction osteogenesis has been extensively explored and debated since it eventually results in a reduced treatment period and related risks for patients who have posttraumatic limb deformities. It has been suggested that lengthening with high distraction rates, as well as an increase in the daily quantity of distraction, be used [16, 17]. One of the methods has studied a rate of 3 mm/day of the distraction with monofocal limb lengthening to investigate whether large amounts of daily lengthening would impair function. The findings suggested that high daily lengthening rates resulted in compensatory energy turnover alterations in the muscle, which were adequate to prevent catabolic processes from occurring in the muscle.

Multiple osteotomy sites in the same bone segment have been recommended for bifocal or multifocal lengthening, as well as an increase in the amount of daily bone distraction by executing simultaneous distraction at osteotomy sites in the same bone segment [8, 18]. There is strong evidence that double-level bone transport can greatly minimize treatment time in the restoration of tibial defects and prevent further procedures as well as associated problems [19]. Double-level bone transport, according to Yushan et al., may result in shorter lengthening indexes and better functional results than bifocal bone transport [20]. Additionally, Borzunov et al. found that the bone lengthening time in the multilevel group may be shortened by 2.5 times [8]. Bifocal bone lengthening potentially produces twice the amount of regenerated bone than monofocal bone lengthening. Consequently, the tissue tension is twice as great since soft tissues at both osteotomy sites do not extend independently. The development of tensile force is dependent on the total lengthening of the segment throughout the day. Muscle contraction occurred in half of the instances in our study, and it was addressed by physiotherapy. It has been indicated by Nayagam that bifocal limb lengthening might help to avoid the possible deformities that can arise with monofocal lengthening when limb length discrepancies are excessive. However, due to the excessive soft tissue stress and potential pain issues associated with bifocal limb lengthening, it should be performed in noncongenital individuals with limb length discrepancies greater than 5–6 cm [6]. In the current series, all patients presented posttraumatic limb length discrepancies, with a mean preoperative LLD of 69.2 ± 6.2 mm.

Preoperative 3-dimensional CT reconstruction clearly identifies the extent of the aberrant femoral marrow cavity, allowing it to be avoided during osteotomy. Fitting the 3D reconstruction models together in image processing tools to accurate lower limb length discrepancies yielded finding the optimum osteotomy site and appropriate position of the screws. Attention to the significance of adequate blood supply to the vascularized bone and neovascularization in the process of bone regeneration, it is necessary to choose an osteotomy level that avoids the nutrient foramina, which are located in the middle third of the diaphysis of the femur. Then, a minimally invasive percutaneous femoral osteotomy was performed using a Gigli saw at predetermined osteotomy sites. During the osteotomy, the periosteum and soft tissue are entirely preserved, however there is no consideration given to the intramedullary circulation.

It is one of the most challenging aspects of limb lengthening and distraction osteogenesis to determine whether the regenerated bone has mineralized sufficiently to remove the external fixator. QCT is a noninvasive substitute for traditional radiography for assessing regenerated consolidation during limb lengthening. Babatunde et al. concluded that there is a good link between QCT scan bone mineral density measures and regeneration maximal torque resistance [21]. For the clinicians, QCT is unique in that it enables high-resolution scanning while also providing quantitative analysis of the regeneration, allowing them to make an objective determination of when to remove the external fixator.

All patients in the present study achieved satisfactory limb length and postoperative bone and functional outcomes. The mean amount of lengthening achieved was 67.5 ± 6.9 mm. In a recent study, Teulières et al. applied motorized intramedullary nails to treat 34 patients with posttraumatic limb length discrepancies. The mean lengthening was 37.5 ± 19 mm for the femur, and the mean healing index was 84.6 ± 62.5 days/cm [3]. They thought bone lengthening with a motorized intramedullary nail device for posttraumatic LLD was considered a safe and effective therapeutic technique. In a previous prospective randomized controlled study, lengthening over a nail and conventional Ilizarov lengthening were compared. The mean duration of external fixation in the lengthening over nail group was 52.2 days compared to 180.4 days in the traditional group. The mean EFIs were 13.2 days/cm and 37.08 days/cm, respectively. The study demonstrated that there was a significant prevalence of deep intramedullary infection with intramedullary nail lengthening compared to standard Ilizarov lengthening, which has fewer complications overall [22].

The ideal time interval of desired lengthening was calculated before operation to predict the external fixation time, which was 34.6 ± 3.1 days on average in the present study. Normally, lengthening of 2–3 cm each month is performed with the unifocal lengthening technique, and 4–6 cm each month with bifocal lengthening. The time required for regenerate consolidation is associated with many factors (osteotomy technique, preservation of the periosteum, rate and rhythm of the distraction, soft-tissue envelope, weight-bearing exercise, etc.), and theoretically speaking, 1 cm lengthening takes 20–30 days for regenerate corticorlization. In the current study, bifocal femoral lengthening was performed with monolateral external fixation. The average duration of external fixation was 180.1 ± 20.2 days while the average EFI was 26.73 ± 1.36 days/cm, which seem not to be better than unifocal lengthening. The main reason is that patients are afraid of treatment failure (considering previous history of operation) and not willing to remove the external fixator in the expected time-point despite the radiographic confirmation of well regenerate consolidation, which potentially extended that time spend on the external fixator. Compared to intramedullary nails, external fixation offers the option of correcting postoperative deformities but also requires precise preoperative planning and postoperative management. With regard to the postoperative complications, the most significant complication in this research was pin tract infection, which was treated with enhanced care and oral antibiotics as determined by the bacterial culture result.

The findings in this report are subject to at least two limitations. First, this study has the inherent limitations of a retrospective study design and a small sample size. Second, an issue that was not addressed in this study was whether the quantified pain and joint mobility have associations with clinical outcomes and the potential to affect the results. To overcome the limitation of statistical approaches, future prospective, multicenter, and large sample studies are needed.

## Conclusions

Precise preoperative planning is a crucial step in the treatment of limb length discrepancy using the Ilizarov technique which could potentially decrease postoperative complications. Bifocal femoral lengthening assisted by preoperative 3-dimensional design in the restoration of posttraumatic LLD is an effective treatment approach.

## Data Availability

All data generated or analyzed during this study are included in this published article.

## References

[1] Brinker MR, Amirian A, O’Connor DP, Laughlin MS (2021). Efficacy of PRECICE nail in treatment of adult patients with posttraumatic femoral leg length discrepancy. J Orthop Trauma.

[2] Axelrod D, Rubinger L, Shah A, Guy P, Johal H (2021). How should we lengthen post-traumatic limb defects? a systematic review and comparison of motorized lengthening systems, combined internal and external fixation and external fixation alone. Eur J Orthop Surg Traumatol.

[3] Teulières M, Langlais T, de Gauzy JS, Rölfing JD, Accadbled F (2021). Bone lengthening with a motorized intramedullary nail in 34 patients with posttraumatic limb length discrepancies. J Clin Med.

[4] Kosuge DD, Pugh H, Timms A, Barry M (2013). Limb lengthening for post-traumatic shortening over a pre-implanted femoral locking plate. J Orthop Trauma.

[5] Hosny GA (2020). Limb lengthening history, evolution, complications and current concepts. J Orthop Traumatol.

[6] Nayagam S (2010). Femoral lengthening with a rail external fixator: tips and tricks. Strateg Trauma Limb Reconstr.

[7] Prince DE, Herzenberg JE, Standard SC, Paley D (2015). Lengthening with external fixation is effective in congenital femoral deficiency. Clin Orthop Relat Res.

[8] Borzunov DY (2012). Long bone reconstruction using multilevel lengthening of bone defect fragments. Int Orthop.

[9] Aarnes GT, Steen H, Kristiansen LP, Ludvigsen P, Reikerås O (2002). Tissue response during monofocal and bifocal leg lengthening in patients. J Orthop Res.

[10] Paley D, Herzenberg JE, Paremain G, Bhave A (1997). Femoral lengthening over an intramedullary nail. A matched-case comparison with Ilizarov femoral lengthening. J Bone Joint Surg Am.

[11] Rozbruch SR, Kleinman D, Fragomen AT, Ilizarov S (2008). Limb lengthening and then insertion of an intramedullary nail: a case-matched comparison. Clin Orthop Relat Res.

[12] Oh CW, Song HR, Kim JW, Choi JW, Min WK, Park BC (2009). Limb lengthening with a submuscular locking plate. J Bone Joint Surg Br.

[13] Guichet JM, Deromedis B, Donnan LT, Peretti G, Lascombes P, Bado F (2003). Gradual femoral lengthening with the Albizzia intramedullary nail. J Bone Joint Surg Am.

[14] Fischgrund J, Paley D, Suter C. Variables affecting time to bone healing during limb lengthening. Clin Orthop Relat Res. 1994;(301):31–37. 8156692

[15] Paley D (1990). Problems, obstacles, and complications of limb lengthening by the Ilizarov technique. Clin Orthop Relat Res.

[16] Stogov MV, Emanov AA, Stepanov MA (2014). Muscle metabolism during tibial lengthening with regular and high distraction rates. J Orthop Sci.

[17] Mizumoto Y, Mizuta H, Nakamura E, Takagi K (1996). Distraction frequency and the gastrocnemius muscle in tibial lengthening. Studies in rabbits. Acta Orthop Scand.

[18] Maffulli N, Lombari C, Matarazzo L, Nele U, Pagnotta G, Fixsen JA (1996). A review of 240 patients undergoing distraction osteogenesis for congenital post-traumatic or postinfective lower limb length discrepancy. J Am Coll Surg.

[19] Catagni MA, Azzam W, Guerreschi F, Lovisetti L, Poli P, Khan MS (2019). Trifocal versus bifocal bone transport in treatment of long segmental tibial bone defects. Bone Joint J.

[20] Yushan M, Ren P, Abula A, Alike Y, Abulaiti A, Ma C (2020). Bifocal or trifocal (double-level) bone transport using unilateral rail system in the treatment of large tibial defects caused by infection: a retrospective study. Orthop Surg.

[21] Babatunde OM, Fragomen AT, Rozbruch SR (2010). Noninvasive quantitative assessment of bone healing after distraction osteogenesis. HSS J.

[22] El-Husseini TF, Ghaly NA, Mahran MA, Al Kersh MA, Emara KM (2013). Comparison between lengthening over nail and conventional Ilizarov lengthening: a prospective randomized clinical study. Strateg Trauma Limb Reconstr.

